# An Experimental Methodology for Automated Detection of Surface Turbulence Features in Tidal Stream Environments

**DOI:** 10.3390/s24196170

**Published:** 2024-09-24

**Authors:** James Slingsby, Beth E. Scott, Louise Kregting, Jason McIlvenny, Jared Wilson, Fanny Helleux, Benjamin J. Williamson

**Affiliations:** 1 Environmental Research Institute, University of the Highlands and Islands, Thurso KW14 7EE, UK; 2 School of Biological Sciences, University of Aberdeen, Aberdeen AB24 2TZ, UK; 3 The New Zealand Institute for Plant & Food Research Ltd., 297 Akersten Street, Port Nelson, Nelson 7010, New Zealand; 4 Marine Scotland Science, Marine Scotland, Edinburgh EH6 6QQ, UK

**Keywords:** environmental monitoring, remote sensing, marine renewables, machine learning, deep learning

## Abstract

Tidal stream environments are important areas of marine habitat for the development of marine renewable energy (MRE) sources and as foraging hotspots for megafaunal species (seabirds and marine mammals). Hydrodynamic features can promote prey availability and foraging efficiency that influences megafaunal foraging success and behaviour, with the potential for animal interactions with MRE devices. Uncrewed aerial vehicles (UAVs) offer a novel tool for the fine-scale data collection of surface turbulence features and animals, which is not possible through other techniques, to provide information on the potential environmental impacts of anthropogenic developments. However, large imagery datasets are time-consuming to manually review and analyse. This study demonstrates an experimental methodology for the automated detection of turbulence features within UAV imagery. A deep learning architecture, specifically a Faster R-CNN model, was used to autonomously detect kolk-boils within UAV imagery of a tidal stream environment. The model was trained on pre-existing, labelled images of kolk-boils that were pre-treated using a suite of image enhancement techniques based on the environmental conditions present within each image. A 75-epoch model variant provided the highest average recall and precision values; however, it appeared to be limited by sub-optimal detections of false positive values. Although further development is required, including the creation of standardised image data pools, increased model benchmarking and the advancement of tailored pre-processing techniques, this work demonstrates the viability of utilising deep learning to automate the detection of surface turbulence features within a tidal stream environment.

## 1. Introduction

### 1.1. Tidal Stream Environments 

Tidal stream environments are coastal areas characterised by fast current flows (often > 1 m/s) [1]. Within tidal stream environments, fast-flowing currents interact with topography and bathymetry to create turbulent features, such as kolk-boils, shears, fronts, and jets, which manifest at the water’s surface [2]. These features provide potential foraging opportunities for marine megafaunal species (marine mammals and seabirds), due to either aggregating or disorientating prey species (fish) [1,3,4]. 

Tidal stream environments are also of key importance to the development of marine renewable energy (MRE) sources, through the extraction of kinetic energy from tidally driven currents [5]. With the UK legislating net-zero greenhouse gas emissions by 2050, tidal energy generation is a continually growing field, with 18 MW of capacity installed since 2008 [6]. 

As it is likely that fauna will encounter MRE devices, and that these devices will alter sediment and flow conditions, further understanding of the potential environmental effects is crucial to enable sustainable development [7,8,9]. In the assessment of the environmental impacts of marine renewable developments, habitat characterisation and animal use have been identified as key knowledge gaps. One such gap is the characterisation of turbulence features within tidal stream environments that provide a potential hydrodynamic habitat [10]. Fine-scale habitat characterisation is crucial in underpinning the understanding of the bio-physical interactions occurring throughout the environment [11]. 

### 1.2. Aerial Imagery

Aerial imagery (particularly using uncrewed aerial vehicles) offers an appropriate method for capturing fine-scale data and can provide increased surveying efficiency compared to land- or boat-based methods [12]. However, optimisation of the method is required to account for the large volumes of data that are produced. Manual processing of imagery datasets can be a bottleneck in large-scale data collection efforts [13]. The effort required to manually review and analyse imagery could therefore negate any gains achieved from using aerial imagery to streamline data collection efforts [14]. With manual processing times increasing concurrently with camera resolution and data volume, the optimisation of post-processing techniques is crucial for the continued use of fine-scale aerial imagery to provide information about the environmental impacts of tidal energy devices. Artificial intelligence offers a method by which post-processing bottlenecks, which are associated with the conversion of imagery into relevant information, could be addressed [15]. This can be achieved via the automatic extraction of meaningful information from imagery by utilising obtained pixel values [16,17]. 

### 1.3. Machine and Deep Learning

Machine learning is a form of artificial intelligence and refers to the general automated approach used to detect meaningful patterns in data [18]. A machine learning algorithm can make autonomous data-driven predictions after being trained on a dataset that allows it to learn how the input relates to a desired output [19]. Within ecological research, supervised machine learning approaches have been highlighted as being appropriate to tackle classification tasks due to their ability to analyse complex non-linear data [20]. While not suitable for carrying out traditional statistical inference or characterising high-order ecological interactions, they can be effective for identifying ecologically important variables for further manual interpretation [21]. 

**Figure 1:**
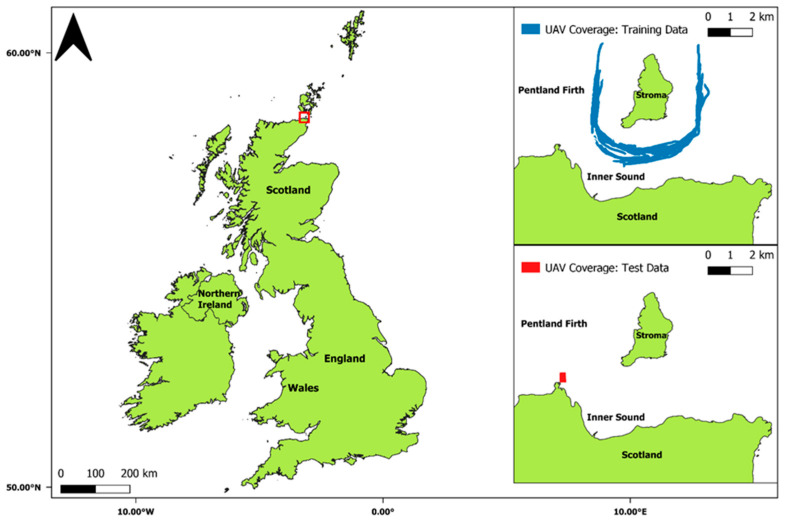
The United Kingdom and the Inner Sound of the Pentland Firth with UAV coverage of training and test datasets displayed.

Over the last decade, deep learning has been a rapidly developing branch of machine learning research [22]. Deep learning skips feature extraction steps with a fully trained model capable of automatically extracting relevant features (Figure 1) [23]. This allows increased generality, with the model being capable of iteratively learning and adapting parameters as exposure to data and their complexity (e.g., changing ecological environments) increases [16]. For object detection tasks, within images, a training dataset containing labelled bounding boxes of objects of interest (OOIs) are the only input requirement [24]. 

While there are various deep learning algorithms, convolutional neural networks (CNNs) are the primary variant in use for object detection and classification within imagery [25]. CNNs have multiple inter-connected layers that allow both a reduction in the complexity of a task, by moving from one layer to the next, and each layer to pull information from those before and after it [26]. Once suitable parameters are selected, the algorithm can be trialled on a test dataset of images, a selection of relevant images withheld from the training process, to allow the quantification of model performance to occur. 

### 1.4. Rationale, Aims, and Objectives

The bottleneck of the post-processing efficiency of imagery data is a key development area for usage in tidal stream environments. Therefore, an examination of the applicability of machine learning methods, to alleviate processing bottlenecks of aerial imagery, is required to assess its potential. To maximise observed performance, it is crucial to utilise a method with the most advanced capabilities. Deep learning (Faster R-CNN) is likely to achieve the highest levels of accuracy due to its increased capability to adaptively tackle challenging object detection tasks within highly variable environmental datasets. 

This proof-of-concept study explores the feasibility of using a Faster R-CNN deep learning model to optimise aerial imagery usage for the detection of turbulence features within tidal stream environments. Aerial imagery (included UAVs) can provide fine-scale (spatial and temporal) assessments of turbulence features, allowing distribution and presence to be characterised, while also providing novel insights, such as area measurements. However, imagery datasets of turbulence features can be large in volume and can take considerable time (days to weeks) to process manually while maintaining a high degree of accuracy. The specific objective of this study is to demonstrate the ability of a Faster R-CNN deep learning model to autonomously detect fine-scale turbulence features (kolk-boils) within the aerial imagery of a tidal stream environment and to enhance the existing manual feature extraction methodologies. Autonomous detection would continue to optimise the survey tools used within tidal stream environments, by addressing the issues of post-processing bottlenecks associated with manual data processing. 

This paper describes the methods used to collect aerial data within a tidal stream environment and the image processing techniques implemented to enhance the quality of the training and test data; finally, it provides a description of the deep learning model implemented in this trial and the processes involved within it. The outputs regarding model performance are presented and discussed in relation to this method’s applicability for autonomously detecting kolk-boil features within a tidal stream environment and enhancing current manual workstreams. 

## 2. Materials and Methods

### 2.1. Study Site

UAV surveys were conducted in the Inner Sound and the surrounding areas of the Island of Stroma, within the Pentland Firth, Scotland (Figure 1). The Inner Sound is a 40-m deep channel, with current velocities generally exceeding 4 m/s and over 6 m/s during peak spring tides [27,28]. These characteristics make the channel a prominent location for tidal energy development, with four 1.5 MW devices installed within the Inner Sound as part of the MeyGen tidal energy project [29]. The area is also a prominent location for marine megafauna transiting and foraging, with many seabird breeding colonies in close proximity [30]. 

### 2.2. Data Collection

The training dataset for the model was created from previous collection efforts involving UAV flights from a moving research vessel (MRV Scotia) within the Inner Sound, near the Island of Stroma, within the Pentland Firth (Figure 1). A DJI Phantom 4 Pro was used due to the multirotor functionality that allows increased stability and positional control [31]. A total of 63 flights were carried out over four-day periods in 2016 and 2018, comprising 15 h and 13 min of total flight time. All the flights conformed to the Civil Aviation Authority (CAA) regulations and recommended best practice guidelines for ecological fieldwork involving UAVs [32,33]. The UAV operations took off and landed from the deck of the MRV Scotia, which was a known height above sea level (10.6 m). By monitoring the UAV GPS readouts, all the surveys were manually flown at constant speed over ground (6 m/s), altitude (70 m), and distance ahead of the vessel (100 m). The flights were carried out almost continuously during daylight hours, during periods of windspeeds ≤ 5 m/s for safety purposes, and against the prevailing tidal current direction to prevent double counting. The flight durations were approx. 10–30 min, with an average of over 200 JPEG images taken during each survey. While the methodologies remained the same across both years, with comparable ground sampling distances (GSDs) (2016: 2.43 cm/px and 2018: 1.96 cm/px) and image overlap (80%), upgrades were made to the camera and UAV specifications between 2016 and 2018. These changes, and the overall UAV methodology, are covered in more detail in Slingsby et al. [34]. 

For the test dataset, eight UAV flights were undertaken from a headland, St John’s Point, at the western periphery of the Inner Sound (Figure 1). The flights were carried out on 18 October 2020 over a one-day period. The UAV flights were preprogramed using the mobile application Pix4Dcapture [35]. The software allowed a consistent survey area, altitude (70 m), and flight speed to be maintained across multiple flights. The UAV survey time was also kept consistent throughout (14 min), unless the operation was halted due to inclement weather conditions. A total of 1333 images were taken, with an image overlap of 80% and a GSD of 1.45 cm/pixels. All the flights were conducted in accordance with CAA regulations and the recommended best practice guidelines for ecological fieldwork involving UAVs [32,33]. 

### 2.3. Data Processing

All the images within the UAV dataset underwent a georectification process before being manually processed to annotate relevant objects of interest. Georectification allowed the registration of images within space (orientation and location), the conversion of image units (pixels to metres), and the calculation of coverage (over ground), as detailed in Slingsby et al. [36]. The images were then processed through a custom graphical user interface (GUI) to delineate kolk-boils and allow precise measurements of feature presence, location, and area. This data annotation is presented in further detail in Slingsby et al. [34,36]. Only images containing kolk-boils were extracted to create a training dataset. Georectification and manual processing occurred using MATLAB software (version R2022b). 

To increase the quality of the training dataset, image enhancements were applied to boost the visibility of the kolk-boils within an image and to allow the potential detection of additional boils missed by previous processing efforts [36]. The image enhancements comprised two main stages: clustering and filtering. The images were first transformed from RGB to monochrome using the rgb2gray function in the MATLAB software (version R2022b) as colour values were not required for the following steps. A k-means clustering algorithm was applied to the monochrome images, using image brightness variance and mean brightness values, to categorise the dataset into four groups based on sea state (Calm, CalmMid, Mid, and Wave). The categorisation allowed the correct filtering process to be applied in the proceeding data processing step. 

**Figure 2:**
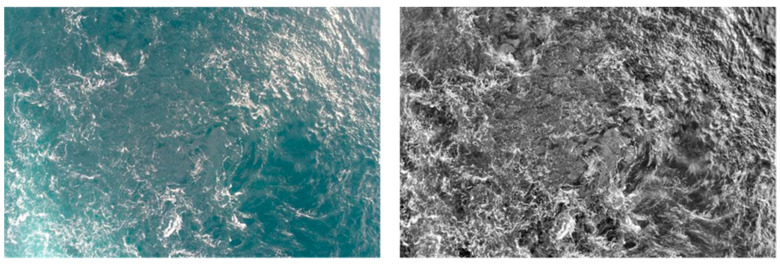
Comparison of (**left**) untreated and (**right**) CLAHE and linear transformed images.

Once image clustering was complete, appropriate filters were applied according to their respective categories. The aim of using filters was to increase the visibility of the kolk-boils and to homogenise the dataset by creating uniformity within the images. Contrast-limited adaptive histogram equalisation (CLAHE) and a per-pixel linear transformation were applied to each image to smooth out the dark and light regions (shadows and glare), respectively, and to increase the uniformity of contrast and brightness within the images to enhance kolk-boil detection (Figure 2). The images were then compiled into the finalised training dataset. While no definitive study on the impact of image enhancements on the model itself was carried out, an increase of 40 detected kolk-boils, compared to previous manual studies without image enhancements [36], was recorded. 

The Microsoft open-source software Visual Object Tagging Tool (VoTT) was used to annotate all the kolk-boils within the training data images, with a ground truth bounding box around each object of interest and a classification given (kolk-boil) [37]. The software provided a JavaScript Object Notation (JSON) file output, which contained the ground truth bounding box coordinates, classification (kolk-boil), and image ID. This information was used, in conjunction with the corresponding images, to create a final, validated, training dataset of 376 kolk-boils. Each image contained kolk-boil features and was used to train an object detection model. 

### 2.4. Deep Learning Model

**Figure 3:**
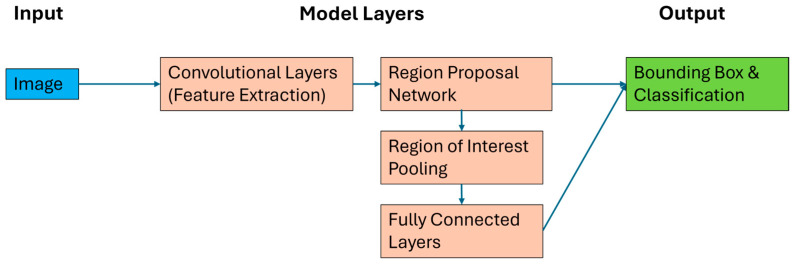
The workflow of Faster R-CNN architecture.

A Faster R-CNN deep convolutional network was used for the object detection model. Faster R-CNN architecture is composed of two modules: a deep fully convolutional network that proposes regions and a Faster R-CNN detector that uses the proposed regions to make a prediction (Figure 3) [38]. The model was run within Python (version: 3.8), using the open-source machine learning framework PyTorch [39,40]. PyTorch allowed the model to be run using Compute Unified Device Architecture (CUDA) Toolkit implementation [40]. The CUDA toolkit enabled the NVIDIA graphical processing unit (GPU) to carry out computations and allowed the training times to be on average 45× faster than central processing unit (CPU)-based work [40,41]. Model training for this study was carried out on a pair of NVIDIA RTX A6000 GPUs, containing 21,504 CUDA cores and 96 GB memory, which were linked using NVLink technology [42]. 

The training dataset and JSON file were input into the model with the evaluation metrics, which were used to iteratively inform the model parameters after the training was complete. To augment the size of the data being fed into the model, each image within the training dataset had a 50% chance of being horizontally or vertically flipped. As is common practice when training CNN models, and to obtain the evaluation metrics, 10% of the dataset was kept aside by the model to be used as a validation dataset [43,44]. Model performance was evaluated using common objects in context (COCO) detection metrics, which provide mean average precision (mAP), recall, and precision values at differing intersection over union (IoU) thresholds (0.50 to 0.95) [45]. As the aim of the study was to detect a boil within an image, as opposed to making accurate predictions of kolk-boils compared to other features, the lowest IoU threshold available of 0.5 (50% overlap) was used. Within the literature, an IoU of 0.5 is deemed to be the “typical” threshold used to determine whether an object has been correctly detected [46]. The evaluation metrics were calculated and used in the determination of the optimal model selection. 

Most of the model parameters were kept at the default with a learning rate (the speed at which the model is capable of learning) of 7×10^−3^ and a weight decay (how quickly the weights change through the model iterations) of 5×10^−6^ (both unitless). These smaller values allow the model to adapt quickly throughout the training process, which is ideal when assessing new OOIs. A batch size of 4 was used and was iteratively determined due to the one class being examined and the processing capability of the GPU used [46]. The number of epochs (the number of passes of the entire training dataset the model has completed) was selected by assessing the evaluation metrics across the models over 10, 50, 75, 100, 125, 135, 145, and 150 epochs. The epoch number of 75 was selected for the final model due to its favourable evaluation metrics and to its being the only variation to have a 100% recall value; this is to be demonstrated within the Results Section [47]. 

**Figure 4:**
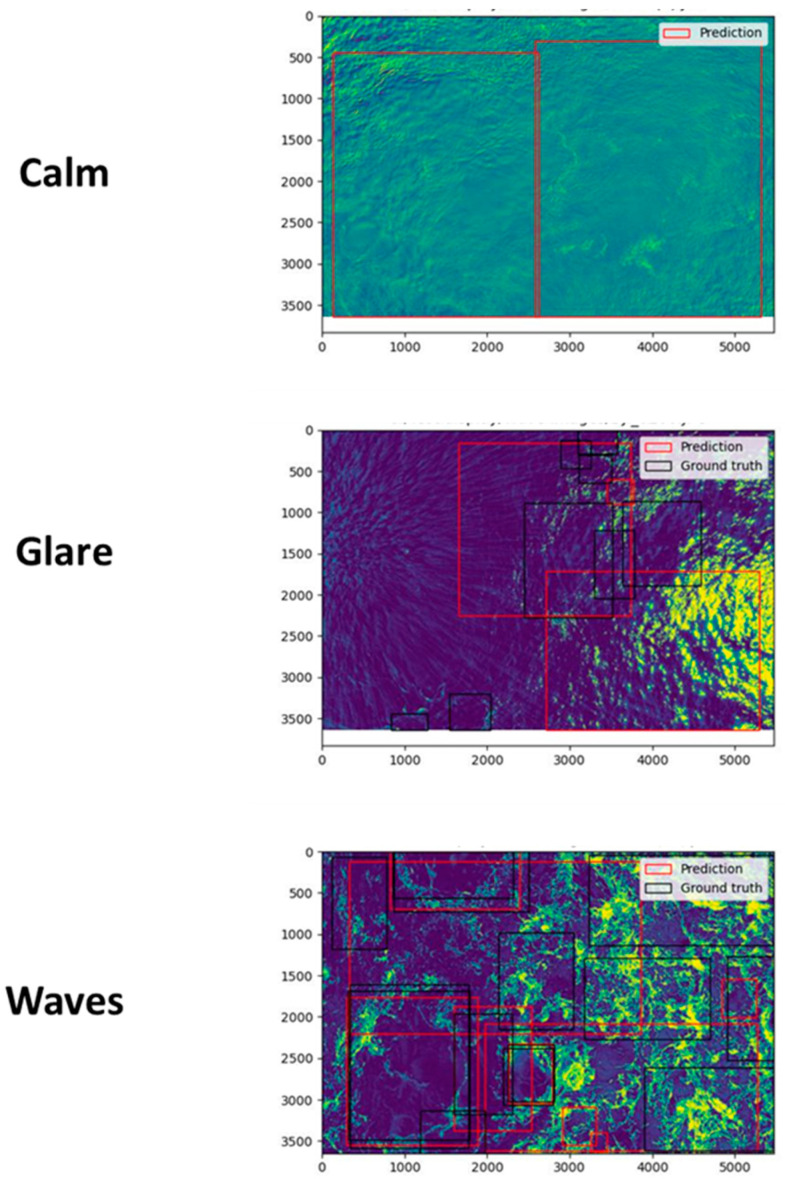
Examples of calm, glare, and wind category images detected by the 75-epoch model.

To test the final model performance, 48 images of kolk-boils were selected from the full test dataset of images. To cover a range of environmental conditions that might be experienced within UAV imagery, the images were subdivided into three groups using the definitions of sea state from the Beaufort scale [48]. Assignments were given to cover the imagery where the environmental conditions were (1) Calm (sea surface is mirror-like, ranging to small, unbreaking wavelets with no sun glare present); (2) Glare (sea surface is mirror-like, ranging to small cresting whitecaps with sun glare present); and (3) Wave (sea surface has waves present in the form of multiple, cresting, wavelets with visible whitecaps and no visible sun glare present) (Figure 4). The model was then used to make a prediction based on each of the chosen images. The model outputs provided a bounding box region for the prediction, a value assigning the class prediction, and a confidence score (%) related to the assigned value. A confidence score shows the probability of an object being correctly detected within an image and is calculated using a logistic regression function based on the corresponding IoU value. Only model predictions with confidence scores above the detection threshold of 0.5 (50%) were included in the study. This value was chosen due to the novel nature of the application of the algorithm (surface turbulence detection) and the limited volume of the training data, meaning that detections may have been missed if the value was initially set too high. 

## 3. Results

The image enhancement techniques allowed the development of the available pool of images containing kolk-boils compared to the untreated efforts. The manual annotation of kolk-boils on untreated images provided 342 usable images for the training dataset, compared to 376 once the above-described image enhancements were applied. This equated to an increase in usable images of 9.94% (34 images).

Average precision (the precision averaged across all intersection over union values) and average recall (the recall averaged over all intersection over union values) provide a metric that can be used for direct comparison between different models. Within this study, average precision and recall were observed to decrease overall as the number of epochs was increased beyond 75, with the 125-epoch variant providing the lowest joint values overall (average precision 10% and average recall 42%) (Table 1). This was also the trend observed for the false positive, false negative, and true positive values. The outlier to this was the 150-epoch variant which had high precision, recall, and low false negatives. However, the number of false positives was high (40), and the percentage of images predicted using this variant was lower than that of the 75-epoch variant (38).

Across all the models trialled, the 75-epoch variant provided the highest average precision (81%) value (Table 1). It also provided the highest percentage of true positives (60) and the lowest percentage values for false positive (40) and false negative (0) kolk-boil detections. This variant also successfully recalled a boil in each of the presented images and had the second highest percentage of correctly predicted images (45).

## 4. Discussion

This proof-of-concept study demonstrates the feasibility of autonomously detecting kolk-boils within UAV imagery of a tidal stream environment. It describes the creation and use of a deep learning algorithm, which is ideal for object detection within complex backgrounds, alongside a tailored workflow (image enhancement and categorisation) to allow the greatest volume of representable imagery to be available during model training. As a result, it provides the innovation of utilising deep learning to detect turbulence features and the ability to complement existing manual methodologies, and it provides an initial pre-processing method to alleviate the impact of different environmental conditions and increase the volume and quality of useable training data to boost model accuracy.

### 4.1. Detection Capability

The model evaluation indicated that a 75-epoch variant offered the highest average precision and recall values and also had optimal percentages of true positive and false negative values compared to the other variants (Table 1). Through utilising this variant, successful detection of kolk-boils was possible in each defined environmental category (calm, wind, and glare) (Figure 4). This highlights the first successful implementation of a Faster R-CNN approach for detecting surface turbulence features within the imagery of a challenging marine environment. 

When assessing detection capability, it is important to consider user requirements and desired outputs. Higher model precision will allow a user to be confident in correctly identifying target classes, at the cost of missing individuals, while increased model recall permits higher detection of objects of interest within an image, at the cost of incorrect identifications [49]. The final model selected demonstrated high false positive values and an average precision value of 81% (Table 1), indicating that further work is still required to create an algorithm geared towards successful positive identification of kolk-boils. However, for this study, the model with the highest average recall value (100%) was selected as it had the capability to find all kolk-boils in an image, and there was the lowest possibility of targets being missed. Although this approach is likely to produce false positive identifications of kolk-boils (40% for the 75-epoch model), the purpose of incorporating automation into this workflow was to lower the processing times of a large imagery dataset (by automatically locating and annotating potential kolk-boils in an image) and to augment an established manual methodology with regard to feature identification and metrics extraction [50]. Further work would be required if this automated approach was to be applied as a sole method for practical kolk-boil feature detection and extraction. 

Even though the model recall was high, the detection of turbulence features within an environment displaying a unique range of environmental conditions creates a complex challenge. Although image enhancement methods can help to highlight turbulence features, the contrast between subject and background and the visual characteristics (e.g., shape and colour) are not consistent compared to studies utilising deep learning to detect megafauna within a marine environment [51]. 

Inconsistencies in target characteristics are increasingly noticeable within periods of high wind, which cause an increase in surface waves, where much of the background of an image is going to be very similar to the perimeter of a kolk-boil [34]. As a result, any deep learning approach to detecting turbulence features within a tidally driven environment will have to develop a method capable of detecting objects of interest that not only vary in visual characteristics (e.g., shape and size) but are also within an environment containing a great potential for false positive detections to occur. 

The most recent advances in object detection have been through the implementation of deep learning approaches [38]. Faster R-CNN architecture is at the forefront of this due to its ability to allow adaptive learning to tackle increased volumes of image noise, lack of visual clarity, and variation in chrominance levels [52]. The faster R-CNN approach explored within this work, while not utilising the algorithm’s full potential, highlights the potential of its detection capabilities and displays a strong use case for continuing to incorporate automated detection into studies of turbulence features within marine environments. 

### 4.2. Image Enhancement

Although the purpose of this study was not to offer a performance indicator between untreated and treated images of a tidal stream environment, it does highlight the importance of a complete image processing methodology, incorporating appropriate image enhancement techniques, when approaching an object detection-based task in the marine environment. Once the image enhancements were applied within this study, there was a 9.94% increase in useable images, and therefore data availability, for the training process of the final deep learning model. Data availability and representativeness are important parameters to consider when incorporating deep learning approaches into environmental studies as they will directly impact the accuracy of outputs [53]. This is crucial for environments which are challenging to survey, such as tidal stream environments, and therefore lack the representative datasets required to feed into automated data interpretation. 

Tidal stream environments are defined by inherently ephemeral turbulence features, such as kolk-boils, that propagate across a diverse range of spatio-temporal scales [2]. While imagery can allow the capture and quantification of such phenomena, the environment itself creates unique challenges when moving to an automated workflow [34]. A common issue when utilising deep learning algorithms, particularly for marine environmental studies, is that the images contain a lot of visual “noise” (e.g., waves, spray, and sun glare) and areas of low contrast, making the differentiation of targets from their surroundings difficult [54]. 

Image enhancement techniques allow the improvement of image quality and visual interpretability [55]. However, this study highlighted that for images of a tidal stream environment, and the marine environment in general, there may not be a “one-size-fits-all” approach, and different automated treatment processes are potentially required on an image-by-image basis. A multi-faceted approach (utilising k-means clustering, CLAHE, and linear transformation) allowed images containing different environmental challenges (e.g., waves, low contrast, and sun glare) to be filtered and processed, via their unique attributes, into a homogenised final dataset. 

While image filtering and enhancements may create a longer workflow, which can be computationally more expensive, they can allow an increase in the quality of a deep learning algorithm training dataset [56]. This was observed in the uses of image enhancement of underwater imagery, where similar environmental issues (blurring and low levels of brightness, contrast, colour quality, and visibility) can hamper an automated detection algorithm’s ability to distinguish objects of interest [57]. However, the proposed image enhancement methodologies, incorporating CLAHE, offered a model accuracy performance increase (up to 3.56%) compared to untreated images [58]. Although this is a different metric to this study (9.94% increase in useable images), both performance indicators helped to highlight the need for the integration of image enhancement techniques into automated object detection workflows, to create increasingly representative training datasets to improve overall model accuracy. 

### 4.3. Using Deep Learning within Tidal Stream Environments

The increase in the complexity of the research questions, and the global challenges, have driven the requirement for a rapid development of technology within ecological research [59]. As renewable energies and associated technologies continue to advance, to meet an increasing energy demand and bring forward a transition to net zero, tidal energy research has seen a similar rapid expansion with key knowledge gaps identified (e.g., resource characterisation, optimal device siting, faunal interactions, and integration within existing coastal management schemes) [60]. The use of deep learning is key to keeping pace with this continued advancement, with these automated approaches now widely used in ecology [61]. Within the marine environment, deep learning methods are already providing an invaluable tool for consistent monitoring, object detection, and estimations of abundance tasks, with the ability to surpass the overall performance of manual efforts in a much smaller timeframe [62]. 

Deep learning approaches incorporating CNNs, as used in this study, are being made increasingly efficient as several architectures are now being pre-exposed to large training datasets of general-purpose imagery and thus have pre-existing weights which can be quickly retrained on specific datasets [61]. The process of utilising a generic dataset as a base for a model and then training it on a more specific one is defined as “fine-tuning” and is far less resource intensive, and complex, than building a custom detection algorithm from the ground up. Testing the suitability of pre-trained CNN architecture and applying fine-tuning would be a suitable next step for the work demonstrated in this study. However, it is heavily dependent on the implementation of CNN-based techniques within marine environments and the provision of standardised datasets for general training. 

CNN-based techniques also outperform other machine learning methods, by up to 30% in some cases, with the deep learning architecture being able to handle complex data patterns more efficiently and detect shapes that are difficult for humans to discern [53,63]. 

With the expanding number of studies within tidal stream environments that utilise large datasets to answer increasingly complex research gaps, the requirement for method automation is becoming a necessity [1,34,64,65]. As a result, tidal stream environments provide an optimal use case to investigate the incorporation of deep learning techniques with the ability to improve current workflows, increase detection accuracies, and increase the viability of using novel methodological approaches. However, applications should take a considered approach when implementing deep learning techniques and make sure that a quantifiable benefit to workflow, or accuracy, can be demonstrated. 

### 4.4. Future Developments

Deep learning has the capability to solve multifaceted tasks within marine research and will play a key role in increasing the efficiency of routine data processing and reducing the amount of manual work associated with large, complex, datasets [53]. However, there are still drawbacks to deep learning use and areas where further development is required. The primary concern for deep learning incorporation into marine research workflows is the lack of available data for annotation to adequately train models [61]. While this can be overcome by artificially augmenting datasets (e.g., flipping, resizing, or rotating), it can only be carried out a finite number of times, meaning that primary labelled examples are still preferential and required. The development of dedicated image libraries to initially train models before tailoring to specific use cases, is important in helping to tackle this. Within marine benthic ecology studies, dedicated image libraries (FathomNet), and associated labelling software (BIIGLE 2) have already highlighted the importance of creating highly accessible tools and training assets to help scientists answer specific research questions [66,67]. For deep learning approaches to continue to become increasingly mainstream within marine research, and specifically tidal stream environments, the creation of pre-existing image libraries and accessible annotation tools is a key factor in ensuring that their advantages are not outweighed by their complexity. 

As the number of use cases of deep learning within tidal stream environments increases, so will the ability to standardise the benchmarking of specific models and approaches. This is important as it will allow the selection process of a suitable deep learning model to be made increasingly less complex for non-experts [68]. However, it will also provide important insight into the current state of machine learning tools within marine research and allow the assessment of the utilisation of new techniques and their potential limitations and trade-offs in terms of the time expenditure, understanding, and knowledge required for successful implementation [61]. In turn, this will allow the development of increasingly adaptable and accurate deep learning methods capable of tackling the complex challenges that the marine environment, particularly tidal streams, creates for the automated processing of imagery data. 

## 5. Conclusions

This paper demonstrates, for the first time, the utilisation of a deep learning object detection model for autonomously locating kolk-boils within the UAV-derived imagery of a tidal stream environment. It includes the incorporation of pre-processing techniques (k-means clustering, CLAHE, and per-pixel linear transformation) to allow the specific treatment of images, depending on the environmental conditions, and to ultimately increase the detectability of kolk-boils within the imagery (9.94% increase in useable images). The optimal model demonstrated high levels of average recall (100%) and precision (81%); however, it also provided sub-optimal values for false positives (40%). The results highlight that deep learning can provide an invaluable tool for research within tidal stream environments as the complexity and volume of data increases. However, further data collection, a decrease in the complexity of use, the development of dedicated image libraries for general training, and the incorporation of a standardised benchmarking process are key to the continued uptake of deep learning into tidal stream research as more use cases become available. 

## Figures and Tables

**Table 1:** Evaluation metrics of model performance across different epoch numbers with conditional formatting, used with per-column formatting, to indicate better (green) and worse (red) model performance values, with a colour gradient for interim values.

Epoch Number	Average Precision (%)	Average Recall (%)	False Positives (%)	False Negatives (%)	True Positives (%)
10	89	98	38	0	60
50	65	92	35	4	56
75	81	100	40	0	60
100	18	60	15	15	46
117	40	79	33	15	46
121	8	56	17	21	40
125	10	42	8	27	33
145	41	75	25	10	50
150	70	98	40	2	58

## Data Availability

The data underlying this article will be shared on reasonable request to the corresponding author.
